# New Insights into *Clostridium difficile* (CD) Infection in Latin America: Novel Description of Toxigenic Profiles of Diarrhea-Associated to CD in Bogotá, Colombia

**DOI:** 10.3389/fmicb.2018.00074

**Published:** 2018-01-30

**Authors:** Marina Muñoz, Dora I. Ríos-Chaparro, Giovanny Herrera, Sara C. Soto-De Leon, Claudia Birchenall, Darío Pinilla, Juan M. Pardo-Oviedo, Diego F. Josa, Manuel A. Patarroyo, Juan D. Ramírez

**Affiliations:** ^1^Universidad del Rosario, Facultad de Ciencias Naturales y Matemáticas, Programa de Biología, Grupo de Investigaciones Microbiológicas-UR (GIMUR), Bogotá, Colombia; ^2^Posgrado Interfacultades Doctorado en Biotecnología, Facultad de Ciencias, Universidad Nacional de Colombia, Bogotá, Colombia; ^3^Molecular Biology and Immunology Department, Fundación Instituto de Inmunología de Colombia, Bogotá, Colombia; ^4^Hospital Universitario Mayor—Méderi, Bogotá, Colombia; ^5^Fundación Clínica Shaio, Bogotá, Colombia; ^6^Universidad del Rosario, School of Medicine and Health Sciences, Bogotá, Colombia

**Keywords:** *Clostridium difficile*, PCR, qPCR, *In vitro* culture, toxins

## Abstract

*Clostridium difficile* (CD) produces antibiotic associated diarrhea and leads to a broad range of diseases. The source of CD infection (CDI) acquisition and toxigenic profile are factors determining the impact of CD. This study aimed at detecting healthcare facility onset- (HCFO) and community-onset (CO) CDI and describing their toxigenic profiles in Bogotá, Colombia. A total of 217 fecal samples from patients suffering diarrhea were simultaneously submitted to two CDI detection strategies: (i) *in vitro* culture using selective chromogenic medium (SCM; chromID, bioMérieux), followed verification by colony screening (VCS), and (ii) molecular detection targeting constitutive genes, using two conventional PCR tests (conv.PCR) (conv.*16S* y conv.*gdh*) and a quantitative test (qPCR.*16s*). The CD toxigenic profile identified by any molecular test was described using 6 tests independently for describing *PaLoc* and *CdtLoc* organization. High overall CDI frequencies were found by both SCM (52.1%) and conv.PCR (45.6% for conv.16S and 42.4% for conv.*gdh*), compared to reductions of up to half the frequency by VCS (27.2%) or qPCR.*16S* (22.6%). Infection frequencies were higher for SCM and conv.*16S* regarding HCFO but greater for CO concerning conv.*gdh*, such differences being statistically significant. Heterogeneous toxigenic profiles were found, including amplification with lok1/3 primers simultaneously with other *PaLoc* markers (*tcdA, tcdB* or *tcdC*). These findings correspond the first report regarding the differential detection of CDI using *in vitro* culture and molecular detection tests in Colombia, the circulation of CD having heterogeneous toxigenic profiles and molecular arrays which could affect the impact of CDI epidemiology.

## Introduction

*Clostridium difficile* (CD) has become one of the pathogens having the greatest worldwide clinical and economic impact during the last few years (Hung et al., 2015; Nanwa et al., 2015). When CD colonization is accompanied by dysbiosis (frequently caused by using antibiotics), it can lead to a wide range of pathologies of the gastrointestinal tract, ranging from diarrhea to pseudomembranous colitis, toxic megacolon, colon perforations and even patient death (Leffler and Lamont, 2015). CD infection (CDI) has been identified as the most frequently reported healthcare-associated infection (HAI), having 2.8–9.3 per 10,000 patients-day incidence rates concerning healthcare facility-onset (HCFO) (Evans and Safdar, 2015). Even though CDI's greatest impact has been associated with HCFO, increased CDI community-onset (CO) has been observed (Chitnis et al., 2013), with incidence rates ranging from 1.3 to 2.7 per 10,000 patients-day (Evans and Safdar, 2015). Such scenarios have been reported in the USA, the UK and other developed countries, where integral CD prevention and control schemes have been brought into clinical practice (Balsells et al., 2016). Latin American CDI dynamics have mainly been studied in Chile, Uruguay, Costa Rica, Argentina and Mexico (Balassiano et al., 2012; Martin et al., 2016) and a 3.1 per 10,000 patients-day CDI incidence rate has been identified concerning HCFO (Lopardo et al., 2015); however, circulating strains' CDI frequency and toxigenic profiles remain unknown for this region.

TcdA and TcdB toxin production is the main CD virulence factor; such toxins irreversibly modify GTPases from the Rho/Ras superfamily, thereby inhibiting critical cell signaling routes (Hussack and Tanha, 2010; Rineh et al., 2014). Such toxins are encoded by genes located in a chromosome region of around 20 kbp, constituting the pathogenicity locus (*PaLoc*) (McDonald et al., 2005) and have been identified as the main causes of symptoms (Carter et al., 2012). Some CD strains can produce the binary toxin, a protein member of the binary ADP-ribosylating toxin family involved in destabilizing host cell cytoskeleton; the binary toxin's subunits are encoded by genes located in the *Cdt* locus (*CdtLoc*) (Gerding et al., 2014). High intra-taxa diversity has been described for CD (Munoz et al., 2017), associated with different organizations for these loci; strains ranging from non-toxigenic to hyper-virulent have been found. The latter are characterized by producing severe clinical pictures (mainly in CDI caused by strains producing the three toxins) (Hunt and Ballard, 2013; Elliott et al., 2017) and by having alarming incidence and mortality rates, causing outbreaks having a great impact on different countries (Clements et al., 2010; He et al., 2013).

Clinically, strategies aimed at detecting CDI are also variable. *In vitro* culture using CD-specific chromogenic agars represents one of the most used approaches (mainly in developing countries where economic resources are limited and there are no set procedures for detecting CDI), chromID *C. difficile* agar CDIF (bioMérieux) has been used due to its efficiency regarding CD recovery (Eckert et al., 2013). Detecting CDI by *in vitro* culture is practical and low-cost; however, it has limited sensitivity and requires long periods of time to be performed (74% after 24 h culture, increasing to 87% after 48 h culture period) (Eckert et al., 2013). Some immunoassays targeting the main toxins (TcdA exclusively or TcdA and TcdB simultaneously) have thus been proposed as alternative (Shen, 2012). Even though these tests are characterized by being rapid and low-cost per test, their limitations are related to their low and variable sensitivity as a single test (46–92%, depending on the type of test and the gold standard used) (Burnham and Carroll, 2013; Planche et al., 2013). Immunoenzymatic tests for detecting the antigen glutamate dehydrogenase (GDH) have thus been proposed as alternative; high levels of GDH are produced by all CD isolates, thereby enabling increased sensitivity as single test of up to 94% (Planche et al., 2013). However, as GDH is a constitutive CD enzyme, this type of test does not facilitate differentiating toxigenic strains from those that are not (Wren et al., 2009). Attempts have been made to counter such limitations by modifying the manufacturers' recommended cut-off points or by carrying out multiple tests independently, as this has led to increasing false positive frequency. This is why the medical community does not currently consider immunoenzymatic tests the best option for diagnosing CDI (Burnham and Carroll, 2013).

Polymerase chain reaction (PCR)-based molecular detection strategies have greater acceptance today as they can detect CDI with greater specificity (~100%) and sensitivity (92–97%), providing reliable results in short periods of time (Burnham and Carroll, 2013). Different molecular markers have been used for conventional PCR, including constitutive genes, such as *16S ribosomal RNA* (*16S.rRNA*) (Naaber et al., 2011) or *gdh* encoding GDH (Paltansing et al., 2007), and others aimed at detecting toxigenic strains using regions from *tcdA* and *tcdB* (Burnham and Carroll, 2013). Even though the latter are useful in the clinical area, they are not sufficiently inclusive for molecular epidemiology studies of CDI; recently, this has led to developing schemes using quantitative PCR (qPCR) targeting constitutive genes (mainly *16S.rRNA*) as a tool for detecting and quantifying CDI caused by toxigenic and non-toxigenic strains (Kubota et al., 2014). Further to detecting CDI, the infecting CD strain's toxigenic potential must be identified, meaning that methodologies involving traditional PCRs describing *PaLoc* presence and organization must be used, as they can decipher arrays in this region of the genome (Griffiths et al., 2010). When coupled to molecular tests targeting binary toxin subunit-encoding regions (located in *CdtLoc*) (Stubbs et al., 2000), this will enable an approach to circulating CD strains' toxigenic profiles.

The forgoing, added to CDI diagnosis limitations such as a lack of knowledge and restrictions in developing counties, led to proposing that this study should be aimed at describing the overall HCFO- and CO-associated CD frequency, followed by a description of circulating CD toxigenic profiles, for the first time in South America. This was done by determining frequency of CD detection (any strain) using a set of tests for identifying them by *in vitro* culture and molecular detection, using three molecular tests targeting constitutive markers (one of them being quantitative). All those samples positive for CD were later subjected to the description of toxigenic profiles using a panel of primers aimed at describing the organization of *PaLoc* and *CdtLoc*.

## Materials and methods

### Sample collection

A set of 217 samples of feces from patients suffering diarrhea was collected from September 2015 to April 2017 from two healthcare centers in Bogotá, Colombia: Hospital Universitario Mayor—Méderi and Fundación Clínica Shaio. Inclusion criteria were defined in line with the “Clinical Practice Guidelines for *Clostridium difficile* Infection in Adults” proposed by the Society for Healthcare Epidemiology of America (SHEA) and the Infectious Diseases Society of America (IDSA) (Cohen et al., 2010). This meant including only older patients suffering diarrhea, defined as the passage of 3 or more unformed stools in 24 or fewer consecutive hours. The site of acquiring the infection was classified according to the aforementioned guidelines; the moment of symptom onset was taken as indicator of site of exposure to CDI. A CO patient case was defined as when the episode of diarrhea was presented during the first 48 h following admission to a medical center, while an HCFO patient case was that where diarrhea occurred after the third day following admission.

Considering the lack of a standardized CDI detection strategy in Colombia and the limited conditions and resources available. This study sought to implement a practical scheme with the lowest possible cost, allowing adequate screening of the samples. To this end, a review of the available literature was carried out, including a scheme previously implemented by our group for the processing of stool samples (Sanchez et al., 2017), which was adapted to the requirements of the media manufacturers and commercial kits implemented. In this context, all diarrheic feces samples were collected in sterile recipients with airtight seal (to avoid direct exposure to oxygen) and without transport media (Shin and Lee, 2014). For storage, the samples were placed inside hermetic recipient and stored under refrigeration (2–8°C) until being processed (within the first 72 h following collection). The samples were then transported at the Universidad del Rosario's Microbiology Laboratory (conserving the cold chain), where they were homogenized by mechanical disruption using sterile scrapers, as the initial step for processing them. The manipulation of the sample during this procedure was carried out at high speed in the laminar flow cabin, taking care of not exposing the sample to oxygen for more than 15 s (Brown et al., 2011).

### Ethical statement

This study was approved by the Universidad del Rosario's Research Ethics' Committee (CEI-UR). This research was considered low risk due to Colombian Ministry of Health resolution 008430/1993 criteria stating that experimental interventions cannot be made regarding research subjects. Data concerning patient identification was treated confidentially, in line with Colombian legal and ethical guidelines and according to that expressed by the latest version of the Declaration of Helsinki (World Medical Association). Hospital informed consent was obtained from HCFO patients. No clinical data regarding CO patients was accessed; this meant that the institution' ethics committee granted permission for the anonymous use of samples for medical research, according to current Colombian regulations concerning ethics.

### Detecting CDI by *in vitro* culture

**Figure 1:**
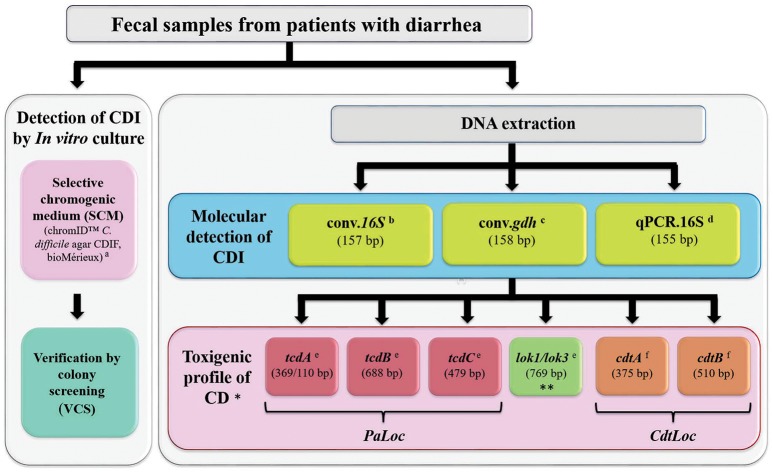
Algorithm used for detecting CDI and describing CD toxigenic profile. ^a^Eckert et al., 2013; ^b^Naaber et al., 2011; ^c^Paltansing et al., 2007; ^d^Kubota et al., 2014; ^e^Griffiths et al., 2010; ^f^Stubbs et al., 2000. ^*^PCR for determining toxigenic profiles were made for all samples having a positive result by at least one of the molecular tests used for detecting CDI. ^**^ Targeting *cdd1*/*cdu1* genes flanking *PaLoc*. A PCR amplification product (using a traditional polymerase) indicated the lack of *PaLoc* in non-toxigenic strains. Amplification sizes relate to those reported in each reference.

The CDI detection algorithm described in Figure 1 was applied to the samples immediately after the mechanical disruption process (within the 15 s limit). The sample was extended by depletion directly over the chromID *C. difficile agar* CDIF (bioMérieux) using a sterile swab. Samples were immediately transferred to an anaerobic jar and incubated under anaerobic conditions (using a GasPak EZ Anaerobe Pouch; Becton Dickinson) for 48 h at 37°C. This culture diagnosis strategy has been reported as being highly sensitive for CDI from symptomatic patients' stools (Eckert et al., 2013). A sample was considered positive by SCM when colonies having the macroscopic morphology described by the manufacturer (gray to black colonies, having an irregular or smooth border) were observed following 48 h incubation. SCM results were confirmed by verification by colony screening (VCS) (Figure 1). Because a possible co-existence of different CD genotypes has been reported in a sample simultaneously (Tanner et al., 2010), from 1 to 7 colonies (depending on the amount of colony-forming units recovered for each sample during the initial step in SCM), were extended on trypticase soy agar (TSA) with 5% sheep blood (Becton Dickinson), and subsequently incubated under aforementioned conditions. VCS was completed by microscopic inspection by routine interpretation by Gram staining, using a smear of the colony on a slide with saline solution. A sample was considered positive by VCS when its morphology revealed typical characteristics expected for CD by detection in culture (gram positive bacillus, occasionally sporulated) (Cohen et al., 2010).

### Control strains

ATCC BAA-1870 (*tcdA, tcdB*, and *cdt* presence confirmed by PCR) and ATCC 700057 (toxinotype *tcdA*-, *tcdB*- and binary toxin gene *cdtB* not amplified by PCR) strains were acquired from the American Type Culture Collection (Manassas, VA). The strains were sown on TSA with 5% sheep blood and incubated, according to the aforementioned culture conditions. Cell biomass was recovered in 1X sterile 300 μL phosphate buffered saline (PBS) until a 4 × 10^7^ cell per mL optical density (OD_600_) was reached. The cell suspension was stored at −20°C until further use.

### DNA extraction

A Stool DNA Isolation Kit (Norgen, Biotek Corporation) was used for extracting DNA from a 300 μL aliquot of the previously homogenized feces samples following the manufacturer's instructions. The aliquot of the sample was obtained just after sowing for SCM, deposited in sealed containers and processed within the maximum time limit established for storage (72 h). The commercial kit used was selected for its high performance for the recovery of DNA from feces, despite the complexity represented by this sample source (Mathay et al., 2015; Sanchez et al., 2017). Extracted DNA was recovered in 100 μL elution buffer included in the kit then stored at −20°C until being used. Regarding control strains, an Ultraclean BloodSpin DNA Isolation kit (MoBio Laboratories) was used for extracting DNA from the cell biomass recovered in 1X PBS, according to the manufacturer's instructions, eluting in the same final volume (100 μL). A NanoDrop2000 (Thermo Scientific NanoDrop Products) was used for spectrophotometric quantification of the DNA extracted from the control strains and then stored at −20°C for later assays.

### Molecular detection of CDI

PCR was used for the molecular detection of CDI; three tests targeting constitutive genes previously reported in the literature were performed independently (Figure 1). Two of these tests were conventional PCR (conv.PCR), one targeting *16S.rRNA* (conv.*16S*) (Naaber et al., 2011) and the other *gdh* (conv.*gdh*) (Paltansing et al., 2007). Table 1A lists the sequence of the primers used in each test. Two independent conv.PCR assays were carried out on a Labnet Thermocycler (Labnet International), using GoTaq Green Master Mix (Promega) at 1X final concentration, with 1 μM of each primer and 3 μL of target DNA at 25 μL final reaction volume. The thermal profiles used for the PCRs were standardized from the conditions proposed by the authors for each primer set (Paltansing et al., 2007; Naaber et al., 2011). Test's amplification conditions were verified using 36 ng DNA extracted from ATCC BAA-1870 reference strain as template. The best thermal profiles for each amplification were identified (Table 1B). Serial dilutions from the same stock of DNA were amplified in duplicate in the conditions described for both tests for determining test limit of detection (LoD), defined as the minimum dilution consistently giving a positive result. After verifying the yield of conv.PCR tests, these were carried out with the DNA extracted from the feces samples. 30 ng of DNA extracted from the reference strains were included as positive amplification controls, 30 ng of *Clostridium perfringens* DNA as control of exclusiveness for clostridia species having the same tropism (gastrointestinal tract) and UltraPure DNase/RNase-free distilled water (Invitrogen) as negative PCR control. PCR products were visualized by horizontal electrophoresis on 2% agarose gels (v/v) stained with SYBR Safe (Invitrogen). Hyperladder V (Bioline) was used as molecular weight pattern.

**Table 1:** Oligonucleotides and thermal profiles used for molecular tests.

**(A)**						**(B)**		
**Scheme**	**Target**	**Oligonucleotide**	**Primer sequence (5'−3')**	**Amplified product**	**Ref**.	**Thermal profiles**		
						**Temperature**	**Time**	**Cycles**
conv.*16S*	*16S.rRNA*	conv.16S.rRNA-F	TTGAGCGATTTACTTCGGTAAAGA	157	Naaber et al., 2011	95°C	5 min	1
						95°C	20 sec	35
						58°C	40 sec	
						72°C	50 sec	
		conv.16S.rRNA-R	CCATCCTGTACTGGCTCACCT			2°C	5 min	1
conv.*gdh*	*gdh*	conv.*gdh-F*	GTCTTGGATGGTTGATGAGTAC	158	Paltansing et al., 2007	95°C	5 min	1
						95°C	20 sec	40
						54°C	40 sec	
						72°C	50 sec	
		conv.*gdh-R*	TTCCTAATTTAGCAGCAGCTTC			72°C	5 min	1
qPCR.16S	*16S.rRNA*	q16S.rRNA-F	GCAAGTTGAGCGATTTACTTCGGT	155	Kubota et al., 2014			
						95°C	30 sec	
						95°C	10 sec	50
		q*16SrRNA*-R	GTACTGGCTCACCTTTGATATTYAAGAG			56°C	1 min	
		q*16S.rRNA*-P	FAM-TGCCTCTCAAATATATTATCCCGTATTAG-BHQ1					
Toxigenic profile	*tcdA*	*tcdA-F*	AGATTCCTATATTTACATGACAATAT	369 (+/+)	Griffiths et al., 2010			
		tcdA-R	GTATCAGGCATAAAGTAATATACTTT	110 (−/+)		95°C	3 min	1
	*tcdB*	tcdB1	TGATGAAGATACAGCAGAAGC	688		98°C	20 sec	35
		tcdB2	TGATTCTCCCTCAAAATTCTC			52°C	40 sec	
	tcdC	tcdC-F17	AAAAGGGAGATTGTATTATGTTTTC	479		72°C	50 sec	
		tcdC-R(+462)	CAATAACTTGAATAACCTTACCTTCA			72°C	5 min	1
	Non-toxigenic	lok1(cdd1)	AAAATATACTGCACATCTGTATAC	769				
		lok3 (cdu1)	TTTACCAGAAAAAGTAGCTTTAA					
	Binary toxin	cdtApos	TGAACCTGGAAAAGGTGATG	375	Stubbs et al., 2000	94°C	10 min	1
		cdtArev	AGGATTATTTACTGGACCATTTG			94°C	50 sec	35
		CdtBpos	CTTAATGCAAGTAAATACTGAG	510		54°C	40 sec	
						72°C	50 sec	
		CdtBrev	AACGGATCTCTTGCTTCAGTC			72°C	3 min	1

*Ref., reference; conv., conventional PCR; Qpcr, quantitative PCR; min, minutes; sec, seconds*.

The third molecular test was also PCR-based, though quantitative (qPCR) (Figure 1); it involved using primers and Taqman probe targeting *16S.rRNA* (qPCR.*16S*) according to previous reports (Kubota et al., 2014), except for the quencher, as TAMRA was replaced by BHQ1 (554 nm maximum absorption), widely recommended for use with the fluorophore FAM (495 nm excitation; 515 nm emission) (Marras, 2006) (Table 1A).

qPCR.*16S* amplification involved using 1X FastStart Universal Probe Master mix (Roche), 200 nM each primer, 200 nM probe (Kubota et al., 2014), 3 μL sample, at 25 μL final reaction volume. Table 1B lists the thermal profiles. qPCR tests were carried out on a CFX96 Real-Time PCR Detection System (BioRad). Amplification efficiency was evaluated by constructing a standard curve from the DNA extracted from a suspension of the ATCC BAA-1870 strain in 1X 300 μL PBS, having OD_600_ equivalent to 4 × 10^7^ cells per mL. An initial 36 ng concentration was used for 10^2^–10^−4^ serial dilutions (in duplicate); these were then used for the amplification. Linear regression analysis of the standard curve results led to determining the coefficient of correlation (R^2^), the Y-intercept and the slope (S) of the logarithmic phase of amplification, as reaction efficiency measurement.

After verifying qPCR.16S test efficiency, this was used for all samples (in duplicate) using 3 μL DNA extracted from feces samples as amplification template. The standard curve was included in each of the test's runs for monitoring test efficiency. The amplification controls previously described for conv.PCR were included during the amplifications. Positive samples' threshold cycle (Ct) was used for calculating the initial amount of DNA copies, comparing this to standard curve Ct (absolute quantification).

### CD toxigenic profile

The toxigenic profiles of samples positive for any CDI molecular detection test were determined by conv.PCR amplification of six molecular markers (Figure 1). Four of these markers fell within *PaLoc* regions, following the scheme proposed by Griffiths et al. (2010); the two remaining regions encoding binary toxin subunits fell within *CdtLoc* (Stubbs et al., 2000). The same amplification conditions described for CDI detection tests by conv.PCR were used for amplifying these markers. Table 1A lists the sequences for the primer sets used for each test. The thermal profiles were reported by the authors and are described in Table 1B.

### Verifying primer performance

*In silico* analysis was also used; this involved using sequences from CD strains' complete genomes, downloaded from PATRIC (Pathosystems Resource Integration Center) website's bacterial genomics database (Wattam et al., 2014). The *C. difficile* 630 (NCBI Taxon ID: 272563, Genome ID: 272563.8) reference genome was used for analyzing the primers used in molecular detection of CDI and for the markers falling within *PaLoc* (*tcdA, tcdB*, and *tcdC*). The *C. difficile* F548 (NCBI Taxon ID: 1232195, Genome ID: 1232195.4) non-toxigenic strain's complete genome sequence was used for analyzing primers targeting *cdd1*/*cdu1* (indicating the lack of *PaLoc*) while the *C. difficile* 2007855 (NCBI Taxon ID: 699033, Genome ID: 699033.6) strain's complete genome sequence was used for analyzing primers targeting *cdtA* and *cdtB*, encoding binary toxin subunits. A first *in silico* analysis phase was aimed at identifying primers' annealing sites on the respective complete genome sequences. A second phase involved a description of the primers' basic characteristics [percent of GC content (%GC) and calculated melting temperature (GC+AT Tm)] and the probability of artifacts occurring during PCR amplification (hairpin loops, dimers, bulge loops and internal loops), both independently as well as in combination for each pair of primers). Gene Runner 3.05 software (http://www.generunner.com) was used for the first two *in silico* analysis components. The BLAST tool was used during a third phase for identifying potential amplification targets.

### Statistical analysis

Descriptive statistics were used for analyzing the results, calculating frequencies and percentages (along with their corresponding confidence intervals) regarding a positive result from the different tests (as categorical variables) and means with their standard deviations (SD) for infection burden (as continuous variable). The frequency of events of interest was expressed considering the total of samples positive for each test, regarding the total of samples collected, overall (complete set of samples) or by population (HCFO/CO). Infection frequency identified in each population using the different tests was contrasted by comparing means between independent populations. qPCR.*16S* results were used for evaluating CDI burden means distribution per population (CO and HCFO), reported as 25 and 75 quartile values (RIQ) regarding absolute DNA amount (ng/μL) and on logarithmic scale. The Mann–Whitney U test was used for analyzing the difference between means for comparing CDI burden means according to outcome (HCFO/CO) as the results did not have a normal distribution. Agreement between the tests used for detecting CDI was evaluated by calculating agreement percentages, accompanied by their corresponding Kappa coefficients (κ), standard error (EE), 95% confidence intervals and *p*-values, as parameter regarding their statistical significance. The tests' Kappa coefficients were compared for determining an overall Kappa coefficient for the tests evaluated here. STATA 11 software was used for analysis, fixing a 0.05 significance level.

## Results

### Primers performance

**Figure 2:**
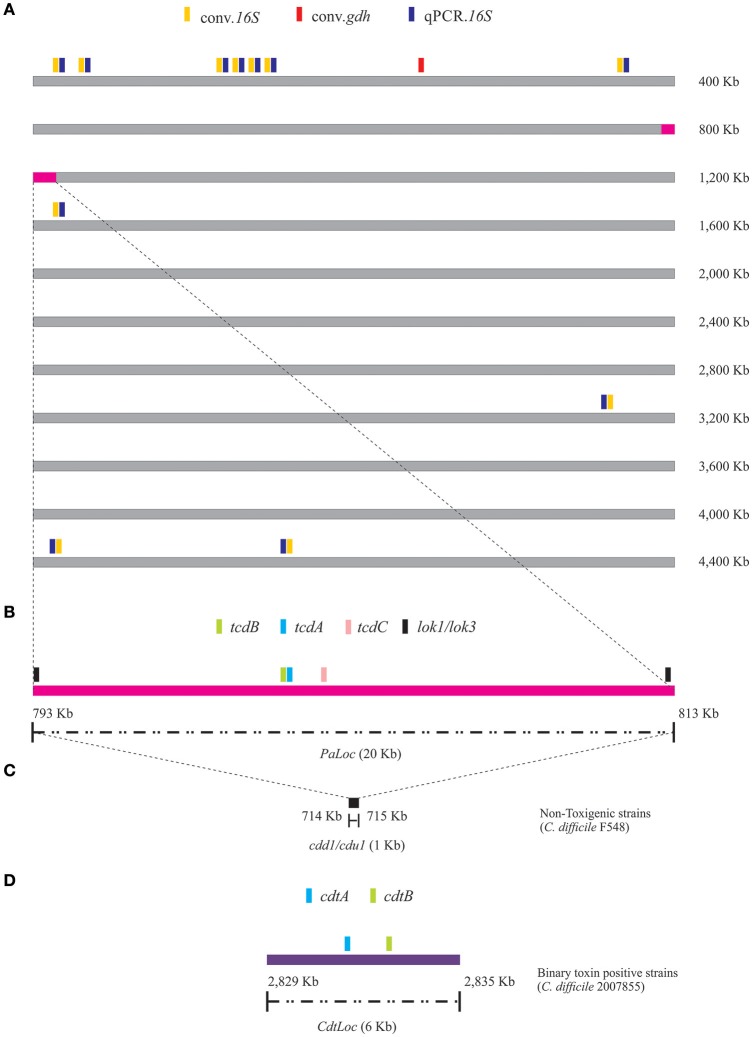
Locating molecular markers which had been predicted by *in silico* analysis. **(A)** Annealing site for the primers used for molecular detection of CDI identified from *C. difficile* 630 (NCBI Taxon ID: 272563, Genome ID: 272563.8) reference strain genome. **(B)** Annealing site for the tests used for determining the toxigenic profiles for samples proving positive for CDI by molecular detection, predicted from *C. difficile* 630 reference strain genome. **(C)** Verifying lok1/3 primers' annealing sites localization on *cdd1*/*cdu1*, from a non-toxigenic strain's genome (*C. difficile* F548; NCBI Taxon ID: 1232195, Genome ID: 1232195.4). **(D)** Identifying annealing sites for primers targeting genes encoding binary toxin subunits, from the genome reported for the strain.

*In silico* analysis of the primer sets used for detecting CDI revealed that those targeting *16S.rRNA* (by conv.PCR and qPCR) had 11 recognition sites in the *C. difficile* 630 reference strain sequence compared to the primer set targeting *gdh* (conv.*gdh*) which only had one recognition site. Aligning the 11 theoretical amplification products extracted from this strain's sequence showed that the gene copies within the genome had one change in three of the eight copies giving 99.4% identity in both cases (156 bp amplification products for conv.*16S* and 155 qPCR.*16S*). This variable position was not located within conv.16S primer set annealing site; however, it was located within qPCR.*16S* reverse primer annealing site, related to the degenerate design in this position. Figure 2A shows the primer sets' annealing sites (predicted from the *C. difficile* 630 reference strain genome); the exact coordinates are given in Supplementary File 1-sheet 1.

*In silico* analysis also enabled verifying that the primers proposed by Griffiths et al. for describing toxigenic profiles based on the *PaLoc* sequence (Griffiths et al., 2010) had a single annealing site within the *C. difficile* 630 strain 20 Kb locus (Figure 2B; Supplementary File 1-sheet 2) and that the region was flanked by *cdd1*/*cdu1*, targeted by lok1/3 primers. The lok1/3 primers' annealing site in non-toxigenic strains was then confirmed from *C. difficile* F548 strain sequence (Figure 2C; Supplementary File 1-sheet 2). The position of the molecular markers targeting the binary toxin (Stubbs et al., 2000) was verified from the *C. difficile* 2007855 strain sequence (Figure 2D; Supplementary File 1, sheet 2).

Gene Runner oligo analysis showed that the primers used in the conv.*16S* test sampled most amplification artifacts, dimers able to generate the direct primer being of greater interest for the procedures. These could happen at probable temperatures (6–46°C) during amplification. Even though some artifacts could have been generated in the other primers used in this study, they were predicted at non-probable temperatures during the procedures (< −20°C). Supplementary File 2 gives oligo analysis results.

BLAST search verification of the most probable amplification targets identified that most primers used in CDI detection tests as exclusively matching CD sequences, except for conv.*gdh* primers which (even though giving better BLAST results with CD sequences) recognized sequences from other species, such as *Vulcanisaeta distributa* and *Arabis alpina* by conv.*gdh*-F and *Lentibacillus amyloliquefaciens* and *Wickerhamomyces ciferrii* by conv.*gdh*-F (query cover was < 90.0% and *E* ≥ 0.250). BLAST analysis of the primers used for describing toxigenic profiles also revealed recognition with other species (query cover was < 90.0% and *E* ≥ 1.00). Supplementary File 3 gives BLAST analysis of primer sequences for primers used in CDI detection tests and Supplementary File 4 those used in describing toxigenic profiles.

### Set of samples

The CDI detection algorithm was used with the set of samples collected (n: 217) (Figure 1). The populations were classified according to the possible source of infection acquisition, following the parameters described in the methodology (Cohen et al., 2010); 36.4% (n: 79) of the samples were HCFO and 65.6% (n: 138) CO. Regarding HCFO, 75.9% (n: 60) came from intensive care unit (ICU) patients and 24.1% (n: 19) from other services' inpatients. Most CO samples were collected from the emergency service (85.5%; n: 118), others from outpatient consultation (14.5%; n: 20).

### Detecting CDI by *in vitro* culture

**Figure 3:**
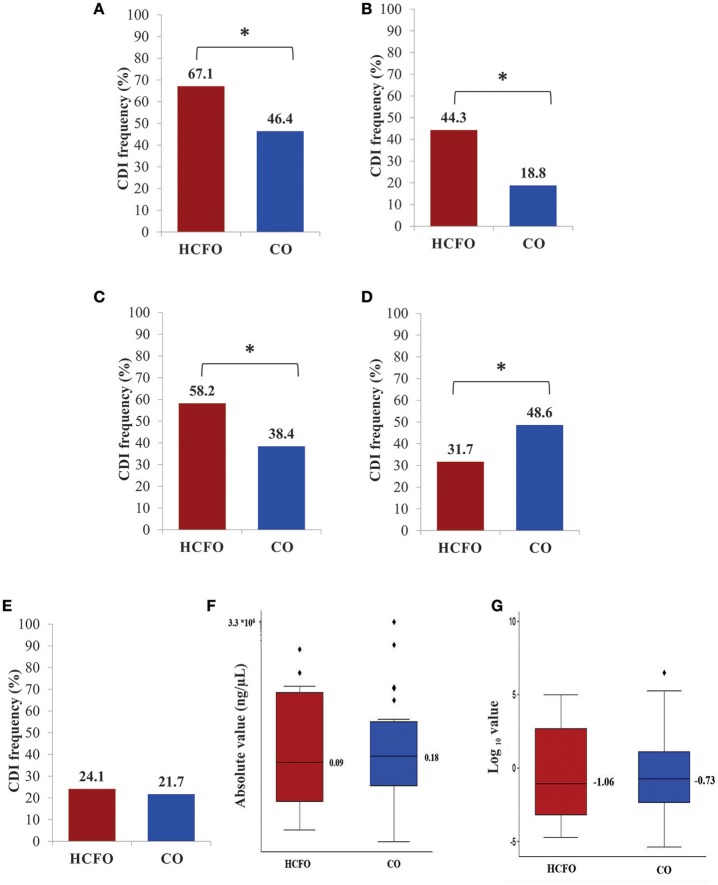
Healthcare facility- or community-onset *Clostridium difficile* infection (CDI) frequencies. **(A)** CDI frequency using SCM (chromID *C. difficile* agar CDIF, bioMérieux). **(B)** CDI positivity determined by SCM culture and confirmed by VCS. **(C)** CDI frequency determined by conv.*16S*. **(D)** CDI frequency determined by conv.*gdh*. **(E)** CDI frequency determined by qPCR.*16S* as qualitative result. **(F)** CDI burden means expressed in absolute values (ng/μL). **(G)** CDI burden means expressed on logarithmic scale. HCFO, healthcare facility-onset (n: 138); CO, community-onset (n: 79). SCM, selective chromogenic medium; VCS, verification by colony screening; conv.*16S*, conventional PCR targeting the *16S* molecular marker; conv.*gdh*, conventional PCR targeting the *gdh* molecular marker; qPCR.*16S*, quantitative PCR targeting the *16S* molecular marker. ^*^The difference between means of positivity having statistically significant difference (*p* < 0.05), according to outcome.

Detecting CDI by SCM gave 52.1% overall frequency (n 113: 45.2–58.9 95%CI). Figure 3A lists infection frequency according to infection source (HCFO or CO). A set of 58 samples (49.6%: 40.2–59.0 95%CI) did not pass VCS screening as they did not grow on plates containing TSA and 5% sheep blood or because their morphology was not as expected (gram positive bacillus with possible presence of spores). Only 59 of the samples initially positive by SCM could thus be verified as VCS positive (50.4%: 41.0–59.85 95%CI), i.e., 27.2% (21.4–33.6 95%CI) overall frequency. Figure 3B describes VCS results per population.

### Using molecular tests for determining CDI frequency

Amplifying molecular markers by conv.PCR was as expected when DNA from control strains was used. The times for each cycle during exponential amplification (5–10 s for each phase) were modified for conv.PCR thermal profiles for improving amplification (obtaining single bands at expected height); conv.PCR LoD determination showed that conv.16S test gave 3.6 × 10^−7^ amplification products and three orders of magnitude lower than those occurring with conv.*gdh* test which gave results lower than 3.6 × 10^−4^. Supplementary File 5 gives the conv.PCR LoD results.

In spite of *in silico* verification, tests on DNA extracted from feces samples identified amplified products having a different size to that expected and sometimes multiple bands. Samples having single bands of different amplification sizes were selected to clarify these findings; Sanger sequencing was then used on them, using the same primers. At least three PCR products were sequenced for each marker. Chromatogram analysis (which included BLAST search) identified bands agreeing with CD in most cases (< 0.000 *E*-values), except for some amplification products matching *Faecalibacterium prausnitzii, Prevotella scopos* and *Burkholderia* sp., having better *E*-values than those found for CD, or even CD results but with *E* ≥1.00. Supplementary File 6 (conv.*16S*) and Supplementary File 7 (conv.*gdh*) show BLAST sequence results. As these bands consistently matched CD, they were considered positive for CDI; all samples had amplification at the heights confirmed by sequencing.

CDI frequency determined by conv.PCR molecular tests was slightly higher for the test targeting *16S*, (positive in 45.6% of the samples; n 99: 38.9–52.5 95%CI) compared to the test targeting *gdh* which was positive for 42.4% of the samples (n 92: 35.7–49.3 95%CI). Figure 3C (*16S*) and Figure 3D (*gdh*) show infection frequency for each population by conventional PCR molecular tests. Analysis of CDI frequency per population showed that the *in vitro* culture approach and conventional PCR targeting *16S* gave higher frequency in the HCFO population; however, conv.*gdh* gave greater frequency regarding positivity for CO, all differences being statistically significant (*p* < 0.05).

Analyzing real-time PCR results (as qualitative outcome—the presence/absence of infection) revealed considerably lower infection frequency than the other two molecular tests (22.6% of positive samples; n 49: 17.2–28.7 95%CI). Figure 3E gives CDI frequency distribution per population.

### CDI burden determined by qPCR

Quantitative analysis of qPCR test results led to determining CDI burden according to outcome (HCFO vs. CO) by comparing means. The results showed that most of the data for the group of patients from HCFO was around 0.086 ng/μL (IQR 0.0006-484.89), being slightly lower than for CO (mean bacterial burden 0.187 ng/μL: IQR 0.004- 13.06) (Figure 3F), however, such differences were not statistically significant (*p* 0.787). Figure 3G gives mean burden results, also represented on logarithmic scale.

A test in conventional format was used as strategy for verifying qPCR.16S results; negative samples having a positive result for some conv.PCR were used for this test. The reaction conditions and thermal profiles described for qPCR.16S were used, but probe volume was replaced by UltraPure DNase/RNase-free distilled water (Invitrogen) and amplification product generation verified by horizontal electrophoresis. Interestingly, verification gave an amplification product at the expected height in most samples.

### Tests comparisons

**Figure 4:**
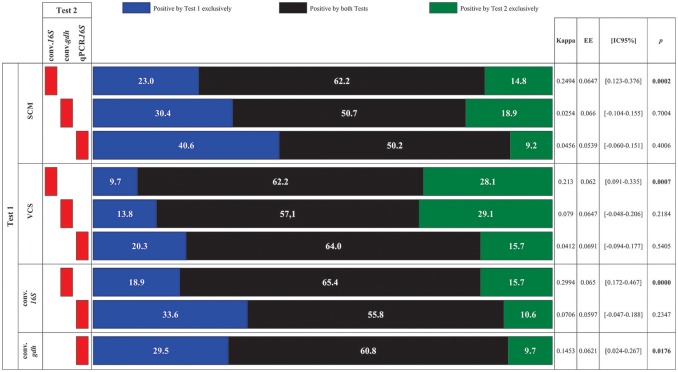
Agreement of tests used for identifying CDI. Test results were compared. Agreement results are expressed in percentages, accompanied by their corresponding Kappa coefficient, standard error (EE), 95% confidence interval [95%CI] and *p*-value. SCM, selective chromogenic medium; VCS, verification by screening of colonies; conv.*16S*, conventional PCR targeting the *16S* molecular marker; conv.*gdh*, conventional PCR targeting *gdh* molecular marker; qPCR.*16S*, quantitative PCR targeting the *16S* molecular marker. Statistically significant *p*-values are highlighted in black.

Analyzing agreement amongst molecular tests used for detecting CDI showed that agreement between tests was limited; conventional PCR targeting the *16S.rRNA* molecular marker (conv.*16S*) gave the highest agreement percentages (statistically significant results) (Figure 4). The best comparison result was con.*16S* with conv.*gdh* (the other conventional test) where agreement was 65.4% (*p* 0.000). The conv.*16S* test had good agreement, even with *in vitro* culture results (SCM and VCS). Interestingly, the results of comparing conv.*16S* to qPCR.*16S* (in spite of having good agreement: 64.0%) were not statistically significant (*p* 0.2347), in spite of targeting the same molecular marker. The other comparison having statistically significant results arose from comparing conv.*gdh* to qPCR.*16S* (60.8% agreement: *p* 0.0176). Figure 4 gives test agreement results. Comparing kappa coefficients between all tests (9 comparisons) gave an estimated 0.1272 overall Kappa coefficient (0.086–0.168 95%CI: *p* 0.0095).

### CD toxigenic profiles

Samples having a positive result by any molecular test (n: 147) were used for amplifying toxin-encoding genes by conventional PCR. A set of 21 samples (14.3%) was toxin typed as negative for all markers evaluated (i.e., by the four molecular markers targeting *PaLoc* and the two detecting binary toxin-encoding genes).

Amplification results led to identifying similar patterns that in the CDI detection tests; in spite of amplification products being obtained at the expected heights (when control strains' DNA was taken as template and from most samples) some products having different sizes to expected size were also identified. The set of lok1/3 primers (targeting *cdd1* and *cdu1* genes) designed to amplify a 769 bp fragment only in the absence of *PaLoc* (as proposed by the authors) (Griffiths et al., 2010) had the most unexpected pattern since amplification size was ≈300 bp in most cases. Sanger sequencing was used for verifying products having unexpected size (analysis including BLAST search); results were similar to what happened for CDI detection tests where most sequences matched CD (< 0.000 *E*-values) and also matched other species. Supplementary File 8 (*tcdA*), Supplementary File 9 (*tcdB*), Supplementary File 10 (*tcdC*), and Supplementary File 11 (lok1/3) give verifying sequences for the markers used for describing toxigenic profiles. All samples giving amplification products having a size matching those verified by sequencing were considered positive for each marker in the toxigenic profile description scheme.

**Figure 5:**
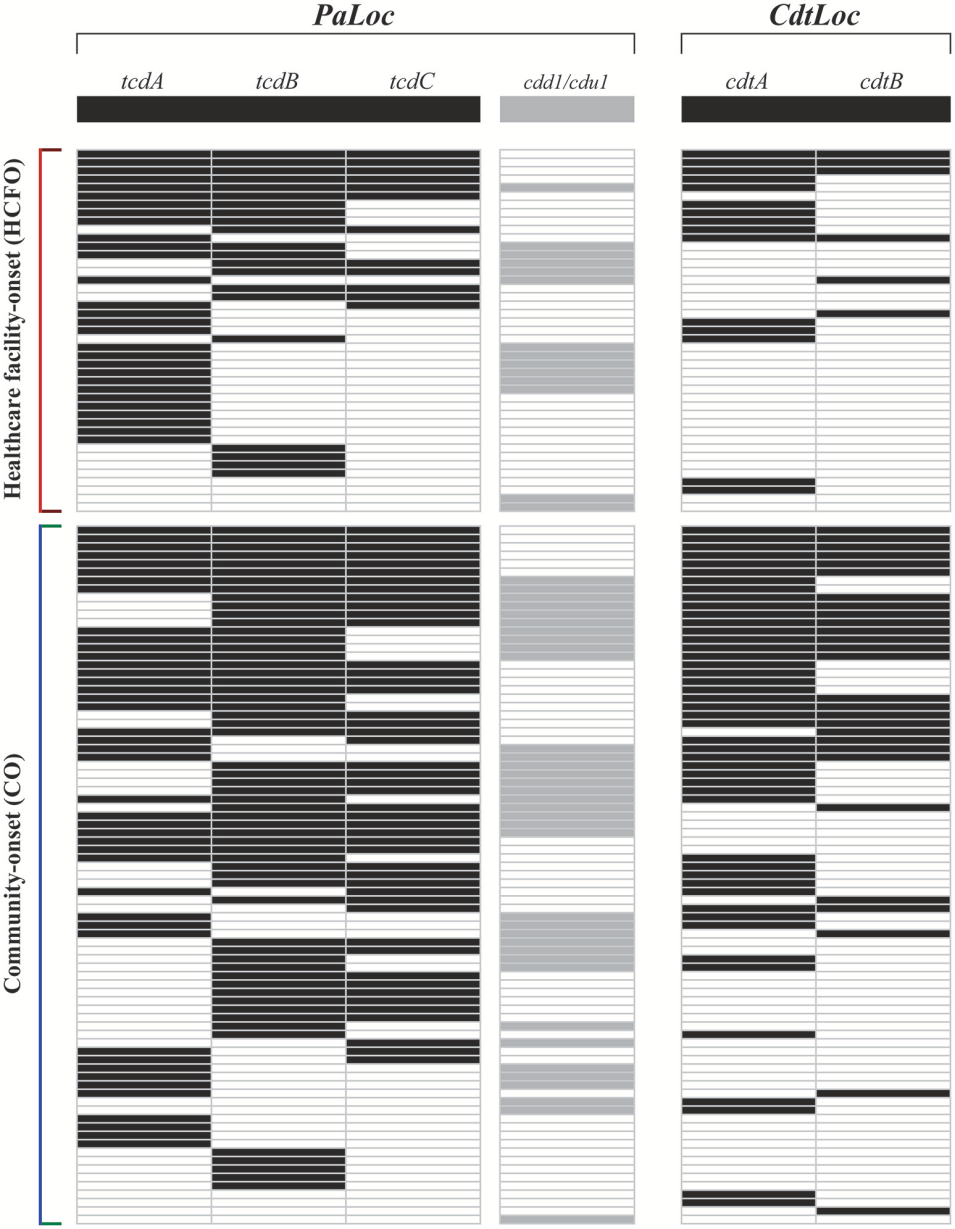
Toxigenic profiles for samples positive for CDI by molecular tests. Toxigenic profiles were detected using primers targeting 4 markers within *PaLoc* and two within *CdtLoc*. The rows represent each sample evaluated. Bars represent a positive result for each marker's amplification.

Describing toxigenic profiles led to identifying circulating strains having toxigenic potential in both study populations (HCFO and CO). The most frequently occurring molecular marker was *tcdB*, being positive in 53.7% (n 79: 45.3–62.0 95%CI) of samples having a toxinotyping result, followed by *tcdA* (49.0% frequency; n 72: 40.7–57.3 95%CI). Another interesting finding regarding the test targeting *cdd1*/*cdu1* was that the mostly positive result were in combination with other PCR targeting *PaLoc* (45 of the 50 positive samples had at least another positive test targeting *PaLoc*). Frequency for genes encoding binary toxin were differentially found, being *cdtA* positive in 42.2% (n 62: 34.1–50.6 95%CI) of samples having a toxigenic profile, while *cdtB* was positive in 23.1% (n 34: 16.6–30.8 95%CI). Figure 5 gives a complete description of the samples' toxigenic profiles. Similar to what happened with the amplification products for both, the CDI detection tests and the *PaLoc* organization description. Amplicons from different expected sizes were found for the two genes of the binary toxin. The results of the sanger sequencing of these products showed a behavior similar to the previous cases [Supplementary File 12 (cdtA) and Supplementary File 13 (cdtB)], ratifying the limitations of these tests to be applied on stool samples.

The results obtained from the samples were informed to the participating health centers, where they were made available to the treating physicians, who took the corresponding therapeutic measures according to the mandatory health plan of Colombia.

## Discussion

The increase in the frequency of CDI in different populations has been attributed to the emergence of hypervirulent strains (Clements et al., 2010; Hung et al., 2015). However, in some cases there exist vast variation in the virulence factors of identified “hypervirulent strains.” This highlights the importance of evaluating the variations in virulence factors and molecular biology of CD (Hunt and Ballard, 2013), with potential for epidemiological monitoring (Nanwa et al., 2015). These facts also confirm the need for screening all types of circulating CD strains and then carrying out the description of their toxigenic profiles, particularly in developing countries where their true impact remains unknown (Allegranzi et al., 2011). Two parameters have been described as key in advancing CDI research: suitable clinical recognition and precise diagnosis (Bartlett and Gerding, 2008).

Regarding suitable clinical recognition, diarrhea (as main CDI-associated symptom) is widely accepted as fundamental inclusion criteria (Cohen et al., 2010) but populations to be analyzed must also be appropriately identified. As exposure to antibiotics is the main factor associated with dysbiosis preceding CD proliferation, individuals in intra-hospital level represent the group at greatest risk due to their frequent exposure to these compounds (Soler et al., 2008), as well as other risk factors favoring progression to more severe clinical view in shorter periods and relapses (Hung et al., 2015). CDI with HCFO (according to SHEA and IDSA classification) constitutes the first target of interest for preventing the impact of CDI. CDI with CO frequency has increased during the last few years (following the same classification criteria) (Chitnis et al., 2013), its impact on health has yet to be clarified, but it could play a key role in disseminating different CD genotypes (Evans and Safdar, 2015).

Differing diagnosis strategies have been proposed for detecting CD and describe the potential impact of colonization at the gastrointestinal level, their use depending on research interests. The present study was aimed at describing CDI frequency in Colombia using an algorithm (Figure 1), designed under the premise of being easy to implement, low cost and suitable on feces samples from patients suffering diarrhea in two populations having the greatest epidemiological relevance: HCFO and CO. The CDI detection algorithm involved *in vitro* culture (Eckert et al., 2013) and molecular approaches, using three tests targeting constitutive markers (Paltansing et al., 2007; Naaber et al., 2011; Kubota et al., 2014) for base line concerning CD molecular epidemiology in Latin America and comparing these tests' usefulness regarding circulating CD in this region.

Concerning *in vitro* culture detection (Figure 1), overall CDI frequency determined by SCM was high (52.1%), being higher in HCFO than CO (Figure 3A). Such results show that although different in-house or commercial media have been used for the storage and transportation of the sample. The processing strategy implemented in this study, designed with the premise of being easy to implement and of low cost, allowed the growth of colonies that coincide with the macroscopic morphology described in the conditions of the manufacturer of the commercial kit, which coincides with other diagnostic strategies implemented with this same premise (Brown et al., 2011; Shin and Lee, 2014). Additionally, the frequencies of infection found by this approach represent a first indicator of CDI's great impact on the populations being analyzed; however, VCS revealed that half the results (50.1%) could not be confirmed, reduced CDI frequency being slightly greater in CO (Figure 3B). Some results failing VCS concerned colonies which did not survive the second round of culture, possibly related to the extreme difficulty of culturing CD (Edwards et al., 2013), but also to colonies which after routine Gram staining inspection had different morphologies to that expected for CD. This represented an indicator of SCM's limited selectivity, matching previously reported findings for this type of approach (Han et al., 2014). Although the verification of microscopic morphology after Gram staining is very broad (including other species and non-toxigenic CDs), it represents a first screening point that can be easily implemented for *in vitro* culture approaches. These findings make it clear that even though *in vitro* culture represents a tool for establishing CD isolates aimed at advancing this pathogen's biology, it is not recommendable for diagnosis. This type of approach must be reconsidered when it is the only alternative for tackling CDI in regions where there is no access to other types of test (using verification strategy described here - VCS). CDI frequency determined by a molecular approach (Figure 1) was greater when comparing conv.16S test (45.6%) to the other two molecular tests; conv.gdh (42.4%) lower frequency, even though having slightly lower overall result (3.2%), was not relevant considering the marked difference regarding the amount of primer annealing sites (11 vs. 1) (Figure 2) and the difference in both logarithmic scales in LoD (Supplementary File 5). qPCR.16S (analyzed as qualitative result) had lower overall CDI frequency (22.6%), accounting for almost half the percentage detected by conventional tests.

Discriminating infection frequency per population showed that CDI with HCFO was greater than in CO in both *in vitro* culture tests (Figure 3A SCM, Figure 3B VCS) and conv.16S test (Figure 3C), having statistically significant differences. Such findings indicate these tests' usefulness for detecting CDI in the population at greater risk. Concerning the conv.*gdh* test (statistically significant and having greater positivity in CO, Figure 3D), this could be used in epidemiological studies for elucidating the impact of CDI with CO. The qPCR.16S test, even though having slightly greater frequency in HCFO, was the only test that did not have a statistical difference when discriminating by outcome (Figure 3E).

Evaluating CDI burden led to identifying more extreme values in the HCFO population, having broader interquartile ranges; the difference between means was not statistically significant (Figures 3F,G). The low infection frequency identified by qPCR.16S, added to amplification products being obtained when primers were used conventionally, indicates that this test's limitation could be in the Taqman probe (either regarding annealing or detecting fluorescence during procedures). Even though *in silico* analysis showed that the probe annealed in a region that seemed to be conserved in sequences reported in databases, real-time fluorescence monitoring during assays (with efficiency indicators, obtained from the calibration curve included in all runs) did not agree with generating amplification products.

Agreement (with corresponding Kappa coefficients) between tests was limited (Figure 4); conv.16S was the only test having statistically significant results when compared to conv.PCR (conv.*gdh*) and *in vitro* culture tests. Regarding qPCR.16S, in spite of targeting the same molecular marker and having the same amount of annealing sites as conv.*16S*, it did not have statistically significant results. Low CDI frequency identified by qPCR.*16S* test, added to a lack of association in statistical analysis (discrimination per population and agreement with other tests), suggested that this test's usefulness for detecting CDI in the population being analyzed should be reevaluated. Such findings are interesting as this test was selected as it had been reported as being efficient for detecting CDI caused by both toxigenic and non-toxigenic strains, even in few copies (Kubota et al., 2014). Such characteristics profile qPCR as a better test than commercial tests exclusive for toxigenic CD, though inversely related to colony forming count for detecting CDI in culture (Kubota et al., 2014).

Considering that CDI impact is defined by its infecting CD toxin-producing capability (Cohen et al., 2010), this study included six molecular markers within its algorithm (Figure 1) which enabled describing the organization of encoding loci for the main toxins (*PaLoc* and *CdtLoc*). This showed that these genes' positivity frequencies were greater than those reported in studies having a similar approach (Han et al., 2014; Putsathit et al., 2017). The most interesting finding concerned the difference in these loci's organization (Figure 5), the most relevant arrays being the different combinations of genes encoding the main toxins (*tcdA* and *tcdB*), since a limited amount of organizations have been reported (Griffiths et al., 2010) and only a couple of years ago reports began to describe this as a loss of *tcdA* in the presence of *tcdB* (Janezic et al., 2015; Monot et al., 2015). On the other hand, although until a couple of years ago, it had been described that *PaLoc* occupies a single position located between cdd1 and cdu1 genes (Van Eijk et al., 2015). The simultaneous amplification of lok1/3 first set (consistently having a different size to that expected, confirmed by Sanger sequencing) with any other *PaLoc* marker (tcdA, tcdB, or tcdC) represents a novel finding regarding the description of toxigenic profiles in Colombia, providing evidence to refute the hypothesis of the location of this locus. These findings coincide with previous reports of different organization of the *Paloc*. However, these were identified in a small number of isolates, after the screening of large sets analyzed and defined as atypical (Monot et al., 2015). Therefore, this study represents the first report of findings of plausible novel *PaLoc* organizations detected directly from feces of patients with diarrhea, revealing new insights of the organization at the molecular level of CD in Colombia. Although later studies must be conducted to elucidate the characteristics of this region, the differences found so far may be due to the characteristics of the mobile genetic element that have been attributed to it, which could favor its mobilization between strains (Mullany et al., 2015), which has also been proven through phylogenomics studies, aimed at evaluating the evolutionary history of *Paloc* among members belonging to different CD Clades (Dingle et al., 2014). In addition to the interesting findings identified in the *PaLoc* organization, a high frequency of positivity for the binary toxin-encoding genes was found in this study, even in the absence of the genes coding for major toxins, which coincides with those previously reported in the literature (Eckert et al., 2015). These findings are of interest, since the CD binary toxin positivity, mainly to *cdtA*-gene, has been associated with severe clinical pictures of CDI (Gerding et al., 2014).

In addition to the arrays described (marker presence/absence), variation in amplification size of the products obtained was identified using different combinations of primers (diagnosis and toxinotyping), some being from other species, including *Faecalibacterium prausnitzii, Prevotella scopos*, and *Burkholderia* sp. This could indicate primer non-specificity when used with DNA extracted from feces samples, representing a sample source for samples having high contaminant content (Siah et al., 2014). However, the most differential sizes found in two approaches (diagnosis and toxinotyping), were confirmed as CD by Sanger sequencing. Such arrays confirmed great evolutionary variation and diversity within this species, whether autonomously acquired (via simple nucleotide polymorphism fixation, short- or long-term intra-genome arrays or gene loss) (Croucher et al., 2014) or by segment exchange (by horizontal gene transfer and recombination events) (Monot et al., 2011). Such capability regarding molecule array fixation in species such as CD could be involved in the rapid dispersion of clinically important loci among genotypes and even among CD lineages (Riedel et al., 2017) as well as affecting the recognition of antigenic epitopes (Du et al., 2014) which, taken together, favors its success as an opportunist pathogen. Such clinically important loci involve toxin production; their atypical organization in CD is being studied (Janezic et al., 2015; Brouwer et al., 2016).

This study's findings represent a baseline concerning the circulation of CD strains having molecular variations in Colombia; added to high CDI frequency, this confirms the hypothesis regarding different stages of CD's epidemic spread worldwide (Martin et al., 2016) and could in the future represent a source of information that allows regulating an adequate screening scheme in developing countries with similar characteristics. However, further work must involve strengthening techniques such as ribotyping and multilocus sequence typing (MLST) for typing and producing information enabling phylogenetic analysis aimed at identifying the Clade to which circulating CD strains in Colombia belong, considering currently accepted classification (Knight et al., 2015). The molecular arrays found to date must also be characterized, this being the best currently available alternative for whole genome sequencing (WGS) which, due to the precision of results and the reduced costs involved, is being increasingly used in pathogen subtyping, describing virulence factors and research regarding outbreaks (Lynch et al., 2016). Adopting such approaches (typing and WGS) involves establishing clinical isolates from positive samples, since all the tests in this research involved using DNA directly extracted from feces samples. As this included a high amount of contaminants (Siah et al., 2014), there may have been multiple CD genotypes (Tanner et al., 2010), thereby affecting these strategies' suitable use.

These research findings should contribute toward knowledge concerning high CDI frequency in Colombia, the limitations of currently available diagnostic tests and molecular variations regarding the markers used for diagnosis and toxin-encoding genes, highlighting the need for CDI prevention and control strategies. It should also aid developing low cost detection tests enabling suitable and opportune identification of CDI that can be used in developing countries, such as Colombia.

## Author contributions

JR and DR-C led the project. MM and MP contributed to definition of experimental approach. MM, DR-C, GH, processed the samples and carried out the tests. MM, SS-DL and JR analyzed the results and conducted the statistical analyzes. MM and JR wrote the manuscript. MP and JR revised the final version. CB, DP, JP-O, DJ, led the clinical component, from the inclusion of samples to analysis of the results at the epidemiological level. All authors reviewed and approved the final manuscript.

### Conflict of interest statement

The authors declare that the research was conducted in the absence of any commercial or financial relationships that could be construed as a potential conflict of interest.

## Abbreviations

CD: *Clostridium difficile*
CDI: CD infection
HAI: healthcare-associated infection
HCFO: healthcare facility-onset
CO: community-onset
*PaLoc*: pathogenicity locus
*CdtLoc*: cdt locus
SCM: selective chromogenic medium
VCS: verification by colony screening
PCR: polymerase chain reaction
conv.PCR: conventional PCR
conv.*16S*: conventional PCR targeting *16S ribosomal RNA* gene
conv.*gdh*: conventional PCR targeting *glutamate dehydrogenase* gene
qPCR: quantitative PCR
qPCR.*16S*: quantitative PCR targeting *16S ribosomal RNA* gene.
