# Supplementary figures and images for: New Insights into Clostridium difficile (CD) Infection in Latin America: Novel Description of Toxigenic Profiles of Diarrhea-Associated to CD in Bogotá, Colombia
Source: Front Microbiol. 2018 Jan 30;9:74. doi: 10.3389/fmicb.2018.00074 (PMC5797639; doi:10.3389/fmicb.2018.00074)

# Limit of detection – conv.PCR

**conv.16S**

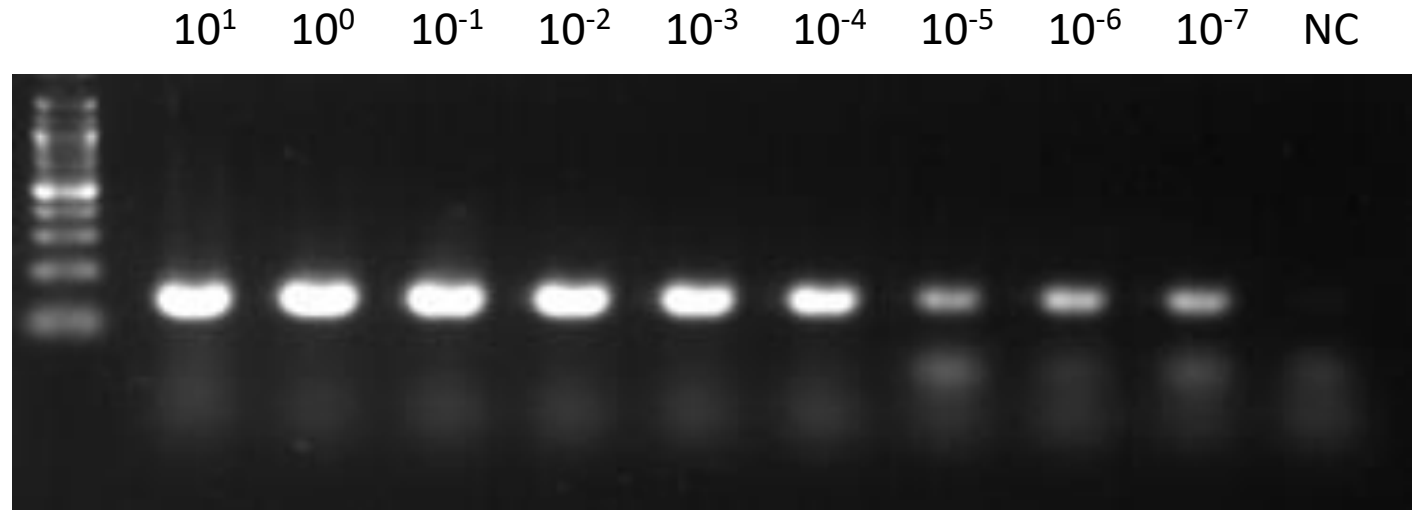

**conv.gdh**

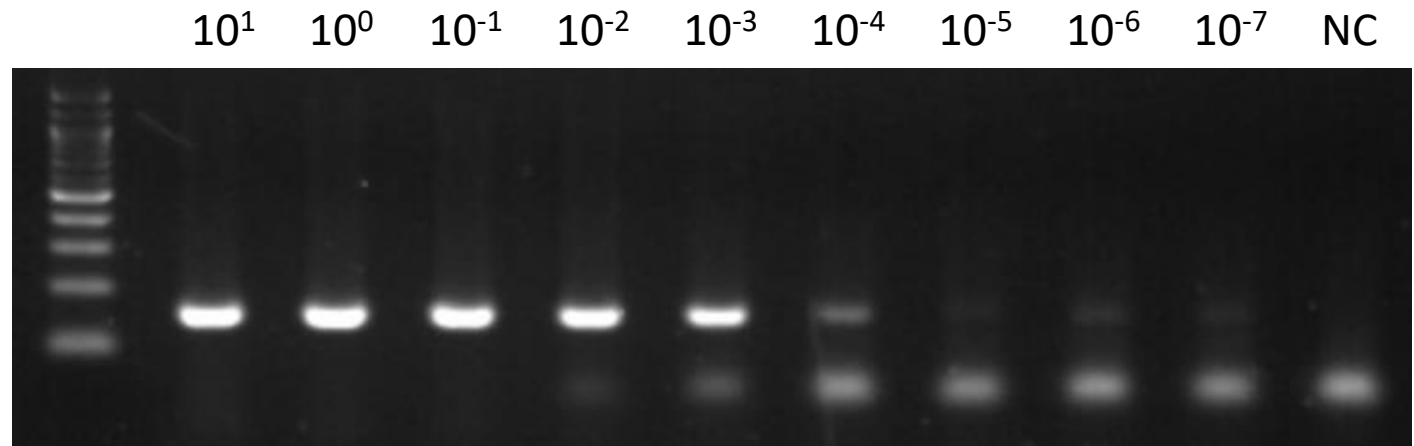

Supplement: Supplementary file 13 [file Table13.pdf]
